# Supplementary material for: Metabolic model predictions enable targeted microbiome manipulation through precision prebiotics
Source: bioRxiv. 2023 Feb 18:2023.02.17.528811. Preprint. [Version 1] doi: 10.1101/2023.02.17.528811 (PMC9949166; doi:10.1101/2023.02.17.528811)
Supplement: 2 [file NIHPP2023.02.17.528811v1-supplement-2.pdf]

bioRxiv preprint doi: https://doi.org/10.1101/2023.02.17.528811; this version posted February 18, 2023. The copyright holder for this preprint (which was not certified by peer review) is the author/funder, who has granted bioRxiv a license to display the preprint in perpetuity. It is made available under aCC-BY 4.0 International license.

11. Supplement

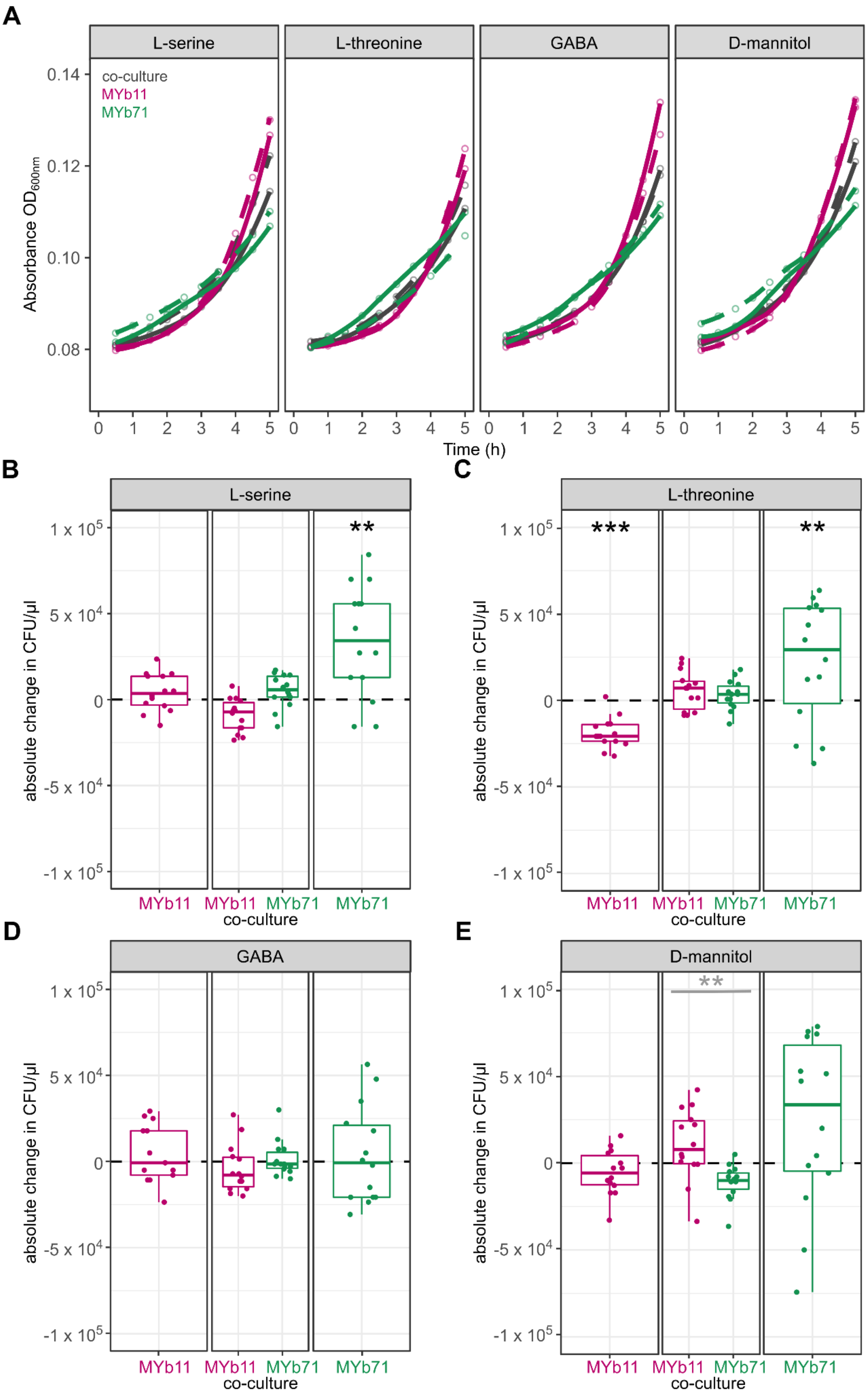

bioRxiv preprint doi: https://doi.org/10.1101/2023.02.17.528811; this version posted February 18, 2023. The copyright holder for this preprint (which was not certified by peer review) is the author/funder, who has granted bioRxiv a license to display the preprint in perpetuity. It is made available under aCC-BY 4.0 International license.

Figure S1: *In vitro* growth of *Pseudomonas lurida* MYb11 and *Ochrobactrum vermis* MYb71 in mono- and co-culture in the presence and absence of four different supplements for 5 h. (A) Growth curves of mono- and co-cultures (gray) of MYb11 (pink) and MYb71 (green) in liquid NGM for 5 h either with 10 mM of L-serine, L-threonine, GABA, and D-mannitol (solid line) or without supplementation (dashed line). **(B, C ,D, E)** Colony-forming units (CFU/µl) in mono-cultures of MYb11 (left) and MYb71(right) or in co-culture (middle) after 5 h of 10 mM of the respective supplement. Shown are boxplots with the median as a thick horizontal line, the interquartile range as box, the whiskers as vertical lines, and each replicate depicted by a dot. Every replicate was normalized by subtracting the non-supplemented median (dashed line) of the respective bacteria. Statistical differences were determined by Wilcoxon signed rank test and are indicated by asterisks (*** $p < 0.001$, ** $p < 0.005$, * $p < 0.05$). Black asterisks indicate statistical comparisons between supplemented and non-supplemented median, gray asterisks indicate statistical comparisons between supplemented medians of MYb11 and MYb71. n = 5-14.

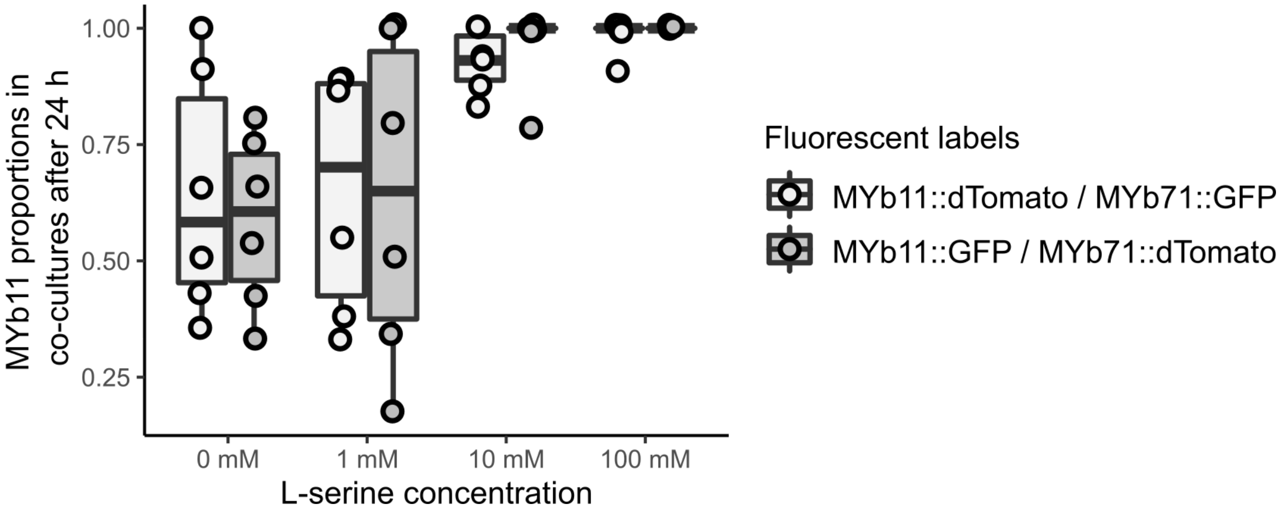

Figure S2. No difference in relative growth rates of differentially labeled bacterial strains. Colony forming units of co-cultures of either MYb11::dTomato/MYb71::sfGFP or MYb11::GFP/MYb71::dTomato were quantified after 24 h of growth in liquid NGM. There was no difference between the two fluorescent labeling systems at any concentration of L-serine (GLM, $p = 0.528$ for main effect of fluorescence; and $p = 0.379$ for interaction between fluorescence and concentration).
